# Graphene Oxide-Doped MgO Nanostructures for Highly Efficient Dye Degradation and Bactericidal Action

**DOI:** 10.1186/s11671-021-03516-z

**Published:** 2021-04-07

**Authors:** M. Ikram, T. Inayat, A. Haider, A. Ul-Hamid, J. Haider, W. Nabgan, A. Saeed, A. Shahbaz, S. Hayat, K. Ul-Ain, A. R. Butt

**Affiliations:** 1 Solar Cell Application Research Lab, Department of Physics, Government College University Lahore, Lahore, 54000 Punjab Pakistan; 2 Physics Department, Lahore Garrison University, Lahore, 54000 Punjab Pakistan; 3 Department of Clinical Medicine and Surgery, University of Veterinary and Animal Sciences, Lahore, 54000 Punjab Pakistan; 4 Core Research Facilities, King Fahd University of Petroleum & Minerals, Dhahran, 31261 Saudi Arabia; 5 Tianjin Institute of Industrial Biotechnology, Chinese Academy of Sciences, Tianjin, 300308 China; 6 School of Chemical and Energy Engineering, Faculty of Engineering, Universiti Teknologi Malaysia, 81310 Skudai, Johor Malaysia; 7 Department of Chemistry, Quaid-i-Azam University, Islamabad, 45320 Pakistan; 8 Department of Physics, Government College University Lahore, 54000 Lahore, Pakistan; 9 Department of Physics, Riphah Institute of Computing and Applied Sciences (RICAS), Riphah International University, 14 Ali Road, Lahore, Pakistan

**Keywords:** Graphene oxide, MgO, Nanorods, Dye degradation, Antimicrobial activity

## Abstract

Various concentrations (0.01, 0.03 and 0.05 wt ratios) of graphene oxide (GO) nanosheets were doped into magnesium oxide (MgO) nanostructures using chemical precipitation technique. The objective was to study the effect of GO dopant concentrations on the catalytic and antibacterial behavior of fixed amount of MgO. XRD technique revealed cubic phase of MgO, while its crystalline nature was confirmed through SAED profiles. Functional groups presence and Mg-O (443 cm^−1^) in fingerprint region was evident with FTIR spectroscopy. Optical properties were recorded via UV–visible spectroscopy with redshift pointing to a decrease in band gap energy from 5.0 to 4.8 eV upon doping. Electron–hole recombination behavior was examined through photoluminescence (PL) spectroscopy. Raman spectra exhibited D band (1338 cm^−1^) and G band (1598 cm^−1^) evident to GO doping. Formation of nanostructure with cubic and hexagon morphology was confirmed with TEM, whereas interlayer average d-spacing of 0.23 nm was assessed using HR-TEM. Dopants existence and evaluation of elemental constitution Mg, O were corroborated using EDS technique. Catalytic activity against methyl blue ciprofloxacin (MBCF) was significantly reduced (45%) for higher GO dopant concentration (0.05), whereas bactericidal activity of MgO against *E. coli* was improved significantly (4.85 mm inhibition zone) upon doping with higher concentration (0.05) of GO, owing to the formation of nanorods.

## Introduction

Water is the most essential component for the survival of living creatures. Large scale industrialization and increasing global warming with the passage of time is declining clean water level rapidly. Earth’s surface is covered with 71% of water where rivers, lakes and fresh ground water accounts for only 0.03% of the total water, which is deemed useful for drinking [1, 2]. 2.5% of water is worthy of consumption, while the rest of the 97.5% is salty water; therefore fresh water supply is in shortage relative to high demands. Approximately, 750 million population of earth is facing clean water shortage and an increased number of micro-pollutants in water have become hazardous for the ecosystem. The world is facing catastrophic consequences caused by environmental and water pollution. Industries are the main source of water pollution owing to discharged wastewater injected with harmful and potentially toxic compounds. Different industries such as textile, paper printing, and food industry use aromatic compounds that can easily dissolve in water causing water pollution and health hazards [3]. Rapid industrialization and urbanization have caused significant environmental pollution through heavy metals that are persistent in the environment. Adverse exposure to heavy metals in the environment is a serious hazard to living creatures [4]. According to WHO, maximum permissible limit of metals in water are, for example, iron (0.1 mg/L), calcium (75 mg/L), magnesium (50 mg/L), copper (1 mg/L) and lead (0.05 mg/L). USEPA reported toxicity profile of heavy metals include lead (damage/fatal to brain), cadmium (kidney damage) and chromium (respiratory tract problems) [5]. The toxic dyes are endangering aquatic lives by blocking sunlight which is necessary for the growth of living organisms. Aquatic species consume these poisonous dyes while these species in turn are consumed by people damaging their health [6].

Various traditional approaches for extracting dyes from wastewater have been suggested, including, evaporation [7], solvent extraction [8], coagulation [9], ion exchange [10], membrane separation [11], physical, chemical and biological techniques [12]. The major problem is that these conventional treatment techniques are expensive when it reaches large scale level. In view of this, researchers have developed various adsorbents such as zeolite, activated carbon, carbon nanotubes, polymers and graphene materials. Adsorption method is extensively used in wastewater treatment to degrade reactive dyes [13–17]. A proper dyes treatment, such as adsorption and catalytic degradation are proposed for dyes removal to improve life quality. Adsorption is cost-effective but catalyst recovery is still a problem that can produce hazardous materials. Catalytic degradation is slightly costly; however it is relatively simple and has the advantage of recyclability [18]. Numerous oxide semiconductors (TiO_2_, ZnO, MgO, Fe_2_O_3_ and WO_3_) have been extensively reported as catalysts for organic dye degradation because of their high chemical stability, toxic-free nature, high activity, and cost benefit.

Among these, non-toxic and cost-effective MgO has been shown to be effective in the areas of adsorption, catalysis for polluted water, superconducting products and antibacterial materials [19–21]. Over the last few years, various nanostructures of MgO, containing nanoparticles, nanoflowers and nanosheets have been fabricated successfully [22]. Recently, MgO with a large band gap of 7.8 eV have revealed a lot of interest because of unique properties: optical, electronic and magnetic [23]. It is widely acknowledged that point defects in crystals such as oxygen vacancies [*V*_o_ (i.e., F^+^-type center, F^+^ center, or F_c_)] may alter material efficiency in bare MgO solids [24]. In addition, MgO has lower density as compared to other metal oxides including cupric oxide, zinc oxide and iron oxide [25]. MgO is an alkaline earth metal oxide with a high pH of zero charge [26], surface area ∼250–300 m^2^/g [27], and zeta potential about − 29.89 mV [28]. Research has found that both particle size and specific surface area are crucial factors that affect the adsorption performance. Hence the surface properties of resulting products are significantly affected by the calcination temperature during synthesis [29–33]. Non-toxic MgO possesses enhanced organic applications as an antibacterial agent for heartburn relief and bone regeneration compared to other Mg compounds as confirmed by U.S. Food and Drug Administration (21CFR184.1431) [34–36].

In the last decade, thick sheet of carbon-based material graphene oxide (GO) has been extensively studied for different applications [37]. Two-dimensional GO nanomaterials demonstrate huge electron mobility, strong chemical stability, wide surface area and high thermal conductivity [38, 39]. Chemical derivative of graphene is GO possessing surface area about 736.6 m^2^/g (theoretical) [40] and zeta potential value of − 113.77 mV [41] containing epoxide, carboxylic and hydroxyl groups. These functional groups result in a negative charge, and hydrophilicity and readily produce GO dispersion in aqueous solution to build a stable suspension [42, 43].

Furthermore, several studies have looked into textile dye degradation for MgO, where MgO was prepared via thermal decomposition and used for methylene blue degradation. 90% of this dye degraded after 180 min [44]. Also, MgO was prepared through a sol–gel method with 15–25 nm particle size, for elimination of 98.3% of dye in wastewater after 300 min [45]. The purpose in this study is to check the influence of GO dopant on MgO, for which various wt. ratios (0, 0.01, 0.03, and 0.05) of GO was doped into MgO using chemical precipitation method. Effects of dopant GO on different characteristics of MgO such as structural, morphological and chemical composition in catalysis and antibacterial action were studied. On the other hand, motivation behind the use of these nanocomposites is to explore the significant use of plasmonic nanomaterials to enhance the antimicrobial as well as catalytic activity of metal oxides.

## Experimental Section

### Materials

Magnesium chloride (MgCl_2_·6H_2_O, 99%), sodium nitrate (NaNO_3_, 98%) and sodium hydroxide (NaOH, 98%) were procured from Sigma Aldrich. Graphite powder (99.5%), sulfuric acid (H_2_SO_4_) and potassium permanganate (KMnO_4_, 99.5%) were procured from Analar.

### Synthesis of Magnesium Oxide and Graphene Oxide

**Fig. 1:**
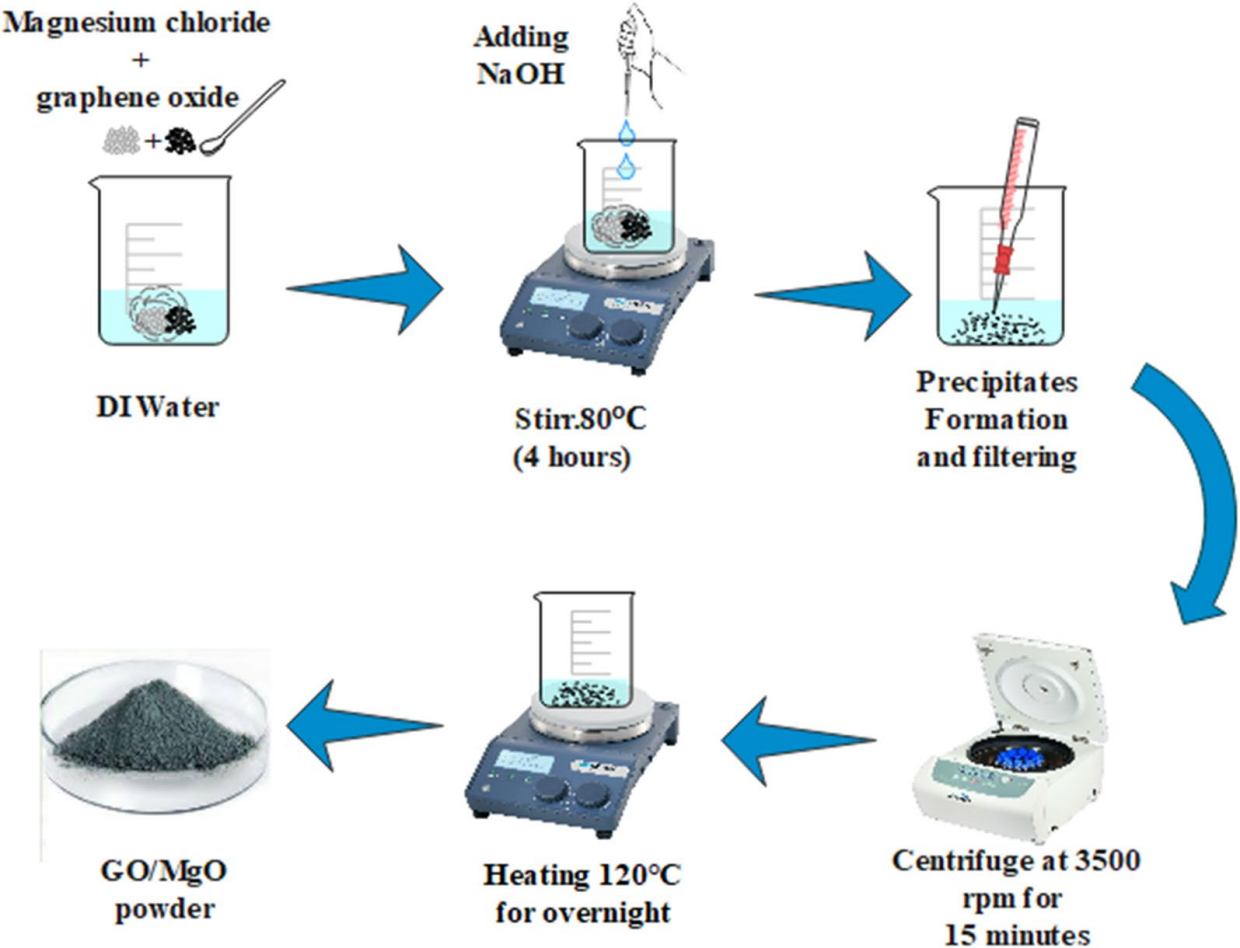
Schematic diagram of the synthesis scheme of GO-doped MgO nanostructures

By following the wet chemical co-precipitation strategy, GO-doped MgO was prepared. The modified Hummer method [46] was used in the preparation of GO. To prepare MgO, desired amount of MgCl_2_·6H_2_O (0.5 M) were stirred into 50 mL of deionized water (DI water) on hot plate. The pH of stirred solution was maintained at 12 using NaOH (0.1 M), which was stirred for 4 h at 80 °C. Stirred solution was centrifuged at 3500 rpm for 15 min subsequently; supernatant was collected and dried at 120 °C for 24 h in the oven. Collected powder was ground using mortar and pestle to achieve fine powders of undoped and GO (0.01, 0.03 and 0.05) doped MgO. The schematic diagram of GO-doped MgO preparation is shown in Fig. 1.

### Catalysis

**Fig. 2:**
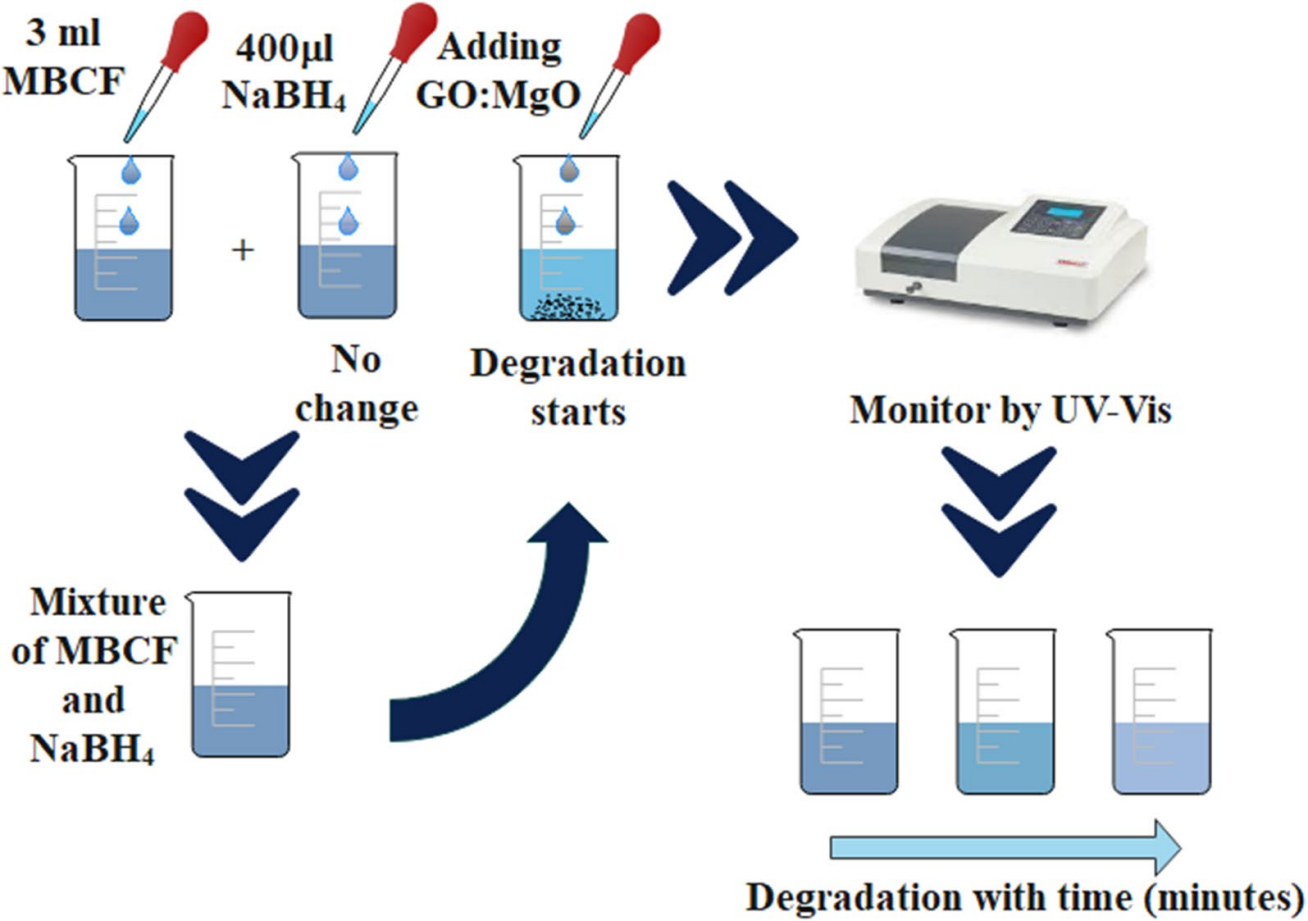
Schematic representation of catalytic activity during dye degradation

Catalytic activity of GO-doped MgO nanostructures was tested against 3 mL MBCF (methyl blue ciprofloxacin) mixed in 400 μL of freshly prepared NaBH_4_ (reducing agent). NaOH and H_2_SO_4_ (400 μL) were added to obtain basic and acidic nature of solution, respectively. After that, 400 μL of doped MgO was added in based solution of reducing agent. Later, blue color of prepared dye (MBCF) started to faint indicating dye degradation of MBCF into Leuco-MBCF (colorless). Samples received from faint solution at various intervals were captured with UV–Vis spectrophotometer in 200–800 nm range as shown in Fig. 2.

### Isolation and Identification of *S. aureus* and *E. coli*

Specimens of mastitis positive sheep milk were retrieved from many veterinary clinics and farms in Punjab and cultured on sheep blood agar (SBA) 5%. Overnight at 37 °C, cultural samples were incubated. Segregated bacterial isolates were purified by streaking on MacConkey and mannitol salt agar (MA and MSA) in triplets maintaining ~ pH 7, respectively. Validation of standard colonies proceeded with gram stain and biochemical analysis (i.e., catalase and coagulase tests).

### Antibacterial Activity

Antibacterial behavior of synthesized material was assessed through well diffusion assay with swabbing 0.5 McFarland of isolated *E. coli* and *S. aureus* bacterial strains on MA and MSA, respectively. A sterile cork borer was used to form wells with a diameter of 6 mm on the MA and MSA plates and distinct concentrations of pure and doped MgO (0.5 and 1.0 mg/0.5 mL) were loaded into each well as minimum and maximum dose in comparison with ciprofloxacin (0.005 mg/0.5 mL) and DI water (0.55 mL) as positive and negative controls, respectively, under aseptic conditions. After an overnight incubation at 37 °C, antimicrobial effectiveness was achieved by calculating inhibition areas in millimeters (mm) using the Vernier caliper.

### Statistical Analysis

A one-way analysis of variation (ANOVA) with SPSS 20 was used to estimate antimicrobial effectiveness in terms of inhibition zone scores (mm).

### Materials Characterization

To identify the crystal structure and phases in the prepared products, the samples were assessed through X-ray diffractometer (model: PAN Analytical Xpert–PRO) using Cu-Kα radiation (*λ* = 1.540 Å) and 2*θ* values from 10° to 85°. Study of attached functional groups was acquired through FTIR (Perkin Elmer spectrometer) used in 4000–400 cm^−1^ range. Absorption spectra were recorded with UV–Vis spectrophotometer (Genesys 10S) in the 200–700 nm range, while using a spectrofluorometer (JASCO, FP-8300), photoluminescence (PL) spectroscopy was performed. Raman Spectra are measured with DXR Raman microscope (Thermo Scientific) using laser based at *λ* = 532 nm (6 mV). Elemental composition was attained via SEM–EDS using INCA EDS software, whereas d-spacing was visualized with the help of high resolution transmission electron microscope (HR-TEM model JEOL JEM 2100F).

## Results and Discussion

**Fig. 3:**
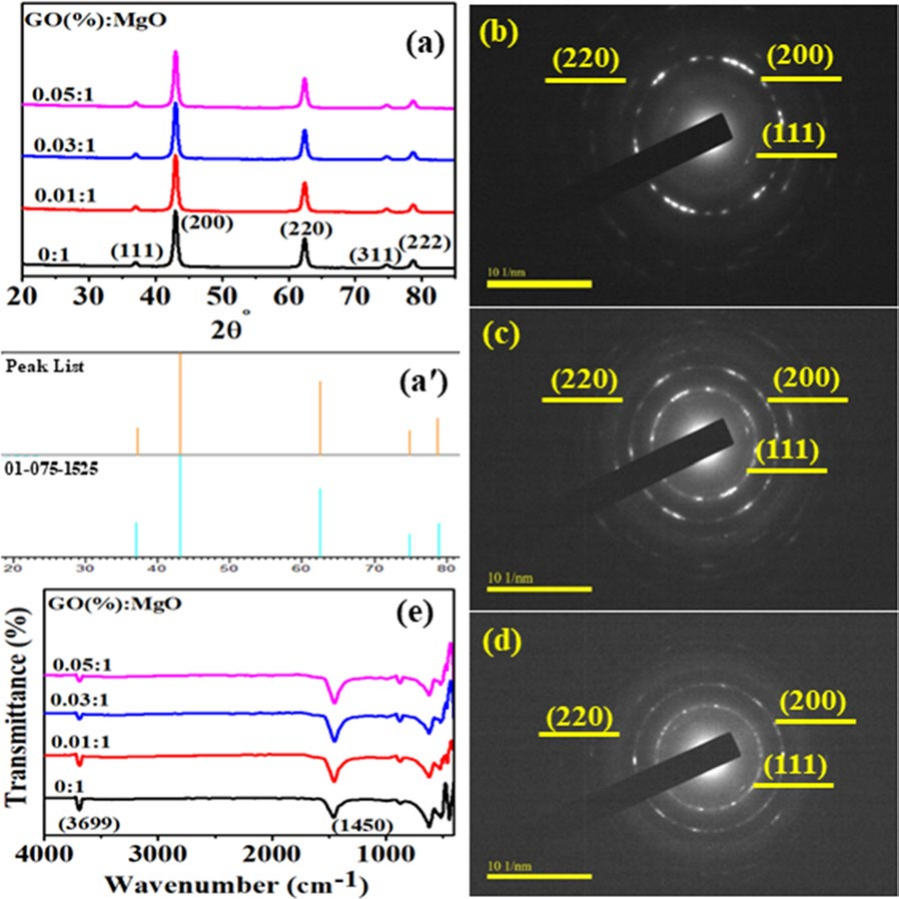
**a** XRD pattern of GO-doped MgO, **a′** Reference code of MgO, **b** SAED pattern of (0:1), **c** GO (0.01:1, **d** (0.05:1), **e** FTIR spectra

XRD of MgO and GO-doped MgO was conducted to identify the crystal structure, size and phase composition in Fig. 3a. Observed peaks at 2*θ*° = 37°, 43.10°, 62.5°, 74.7° and 78.8° were in agreement with (111), (200), (220), (311) and (222) planes confirming that MgO had cubic structure coinciding with (JCPDS 75-1525) [47]. Upon doping, diffraction peaks were identical pointing toward the small content of used dopant GO, which was not detectable. Average crystallite size was calculated from diffraction peaks using the Debye–Scherrer equation, which was found to be 13.28 nm. Calculated d-spacing (0.21 nm) was attributed to (200) lattice plane of cubic MgO. Selected area electron diffraction (SAED) patterns of the samples indicated the MgO crystalline nature (Fig. 3b–d). The bright spot of the concentric rings related well with the XRD planes of MgO [48].

In order to examine the attached functional groups of doped MgO, FTIR was operated in the wavenumber from 4000 to 400 cm^−1^ range as illustrated in Fig. 3e. Transmittance peak around 3699 cm^−1^ in doped MgO was ascribed to characteristic stretching vibration of hydroxyl groups (alcohol) obtained due to reaction between MgO surface and water vapors in air [3]. The reduction in peak intensity with the introduction of GO was observed, which is attributed to dopant sheets wrapped around MgO. The band found at 1450 cm^−1^ was related to asymmetric stretching vibrations of carbonate ions (C–O), while the corresponding bending vibration peaks were noticed at ~ 865 and ~ 867 cm^−1^ [48]. However, decrease in intensity of afore-mentioned bands was observed for GO-doped MgO sample [20]. Band found around 443 cm^−1^ showing the presence of Mg-O characterized stretching vibration [49].

**Fig. 4:**
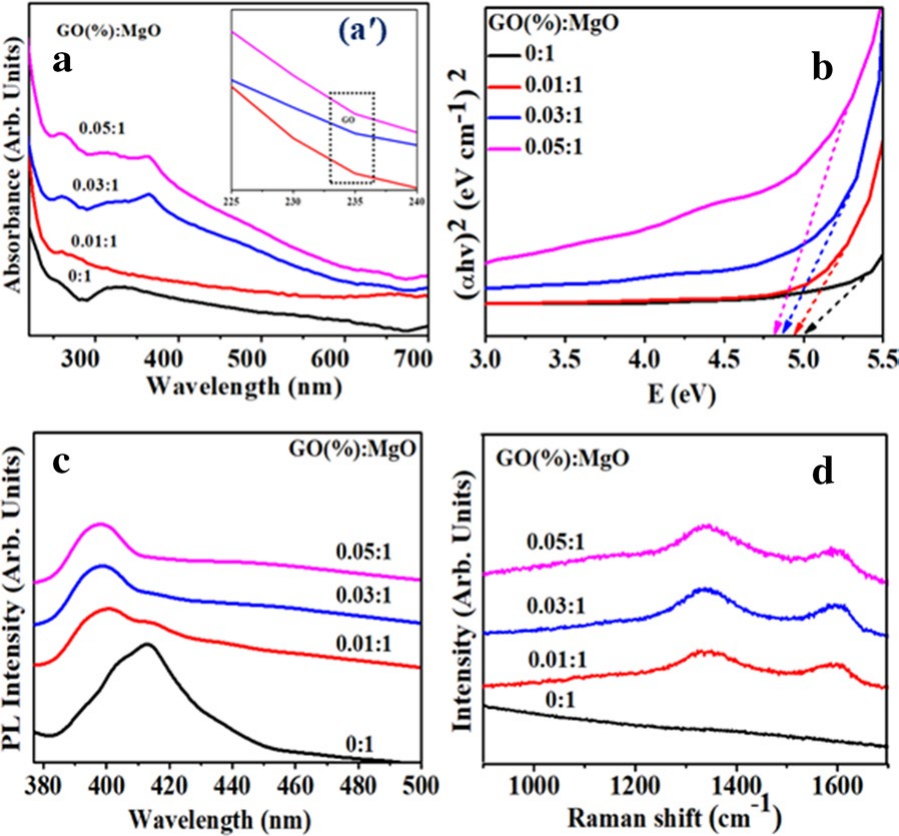
**a** Absorption spectra of different samples of GO-doped MgO, **aʹ** Zoomed absorption spectra of GO-MgO, **b** Tauc plot, **c** PL spectra and **d** Raman spectra of GO-MgO nanostructures

Absorption spectra of dopant-free and doped MgO were collected from 220 to 700 nm range (Fig. 4a). Band absorption for MgO found at ~ 250 and ~ 320 nm can be endorsed to oxygen vacancies (F and F_2_^2+^) centers, respectively. At an excitation *λ* = 250 nm, F center photoionization process is involved, led by the equation F + hυ ↔ F +  + e [23]. Absorption peak of GO was found around 230 nm which can be assigned to *π*–π* transitions of C=C in the amorphous carbon system [50], upon doping absorption increased accompanied by redshift. Band gap decreased slightly (5.0–4.8 eV) with increasing amount of GO in fixed amount of MgO as depicted in Fig. 4b. This redshift suggests morphological effects on crystals having numerous active sites or may be quantum confinement effect [51].

Figure 4c reveals PL emission spectra of doped MgO measured ranging from 377 to 500 nm with an exciting *λ* of 350 nm. To determine the recombination efficiency of charge carriers (migration) and efficacy of trapping, PL was utilized. PL emission peak at 414 nm results from the defect band transition due to energy levels produced with various F (oxygen ion vacancy) type anion vacancies [52]. PL emission of GO is usually attributable to electron–hole recombination from adjacent confined electronic states to broad-range valance band (VB) and bottom of conduction band (CB). In atomic structure, emission mostly generates from electronic transitions among the non-oxidized carbon region (–C=C–) and edge of oxidized carbon atom region (C–O, C=O and O=C–OH) [53]. GO-MgO peaks revealed blueshift and exhibited low intensity, which indicated reduced rate of recombination that might be due to transfer of electron from higher energy level to new generated states.

Surface structure and disorder of fabricated samples were analyzed via Raman spectroscopy as presented in Fig. 4d. The control sample spectrum of Raman did not showed any characteristic peak in 100–1600 cm^−1^ region, which suggests low phonons of MgO scattering intensity [54, 55]. In case of doped samples, bands located around 1338 cm^−1^ (D band) and 1598 cm^−1^ (G band) confirmed presence of GO in the sample [56]. D-band is assigned to defect in sp^3^ carbon (C) atoms and G-band is arising from E_2g_ phonon scattering (1st order scattering) of *sp*^2^ C atoms. Intensity enhancement of D and G peaks is obtained with increased doping concentration. Moreover, intensity ratio (*I*_D_/*I*_G_) of D and G band indicated disorder degree of *sp*^2^ C domains [46].

**Fig. 5:**
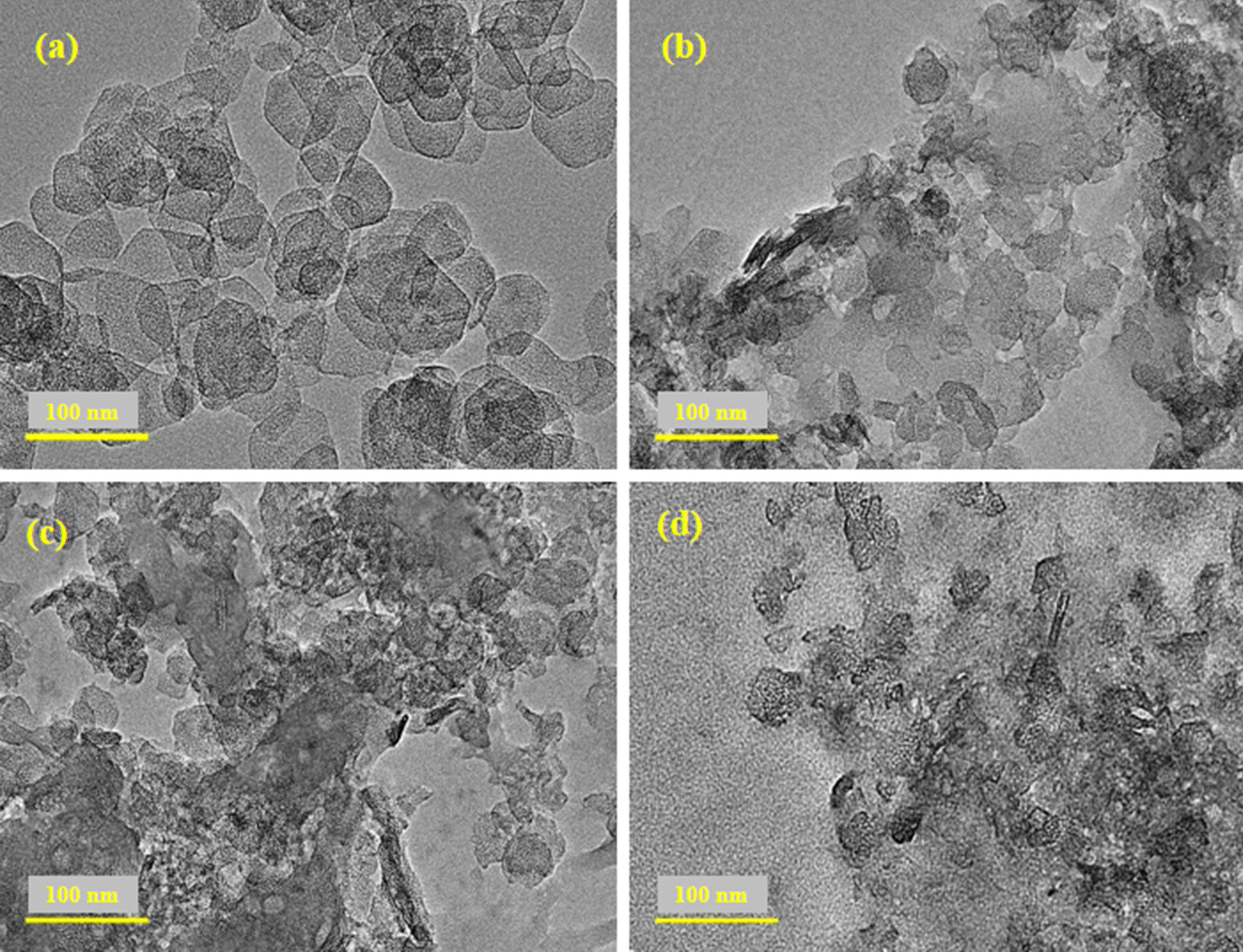
**a**–**d** HR-TEM images of various concentrations of GO-doped MgO (0, 0.01, 0.03 and 0.05), respectively

To confirm the crystal structure and morphology of doped MgO, HR-TEM was employed (Fig. 5). HRTEM image (5a) of dopant-free sample has agglomerated hexagon shaped nanostructure. With incorporation of GO, hexagon morphology of nanostructure were merged with GO nanosheets and formation of rod-type structure in the presence of hexagons was observed. The aggregation increased with increasing amount of dopant in MgO where rods and hexagon morphology of nanostructures were found. It is noteworthy that diameter of hexagon nanostructure decreased with higher concentration of GO confirming the interaction between MgO and GO.

**Fig. 6:**
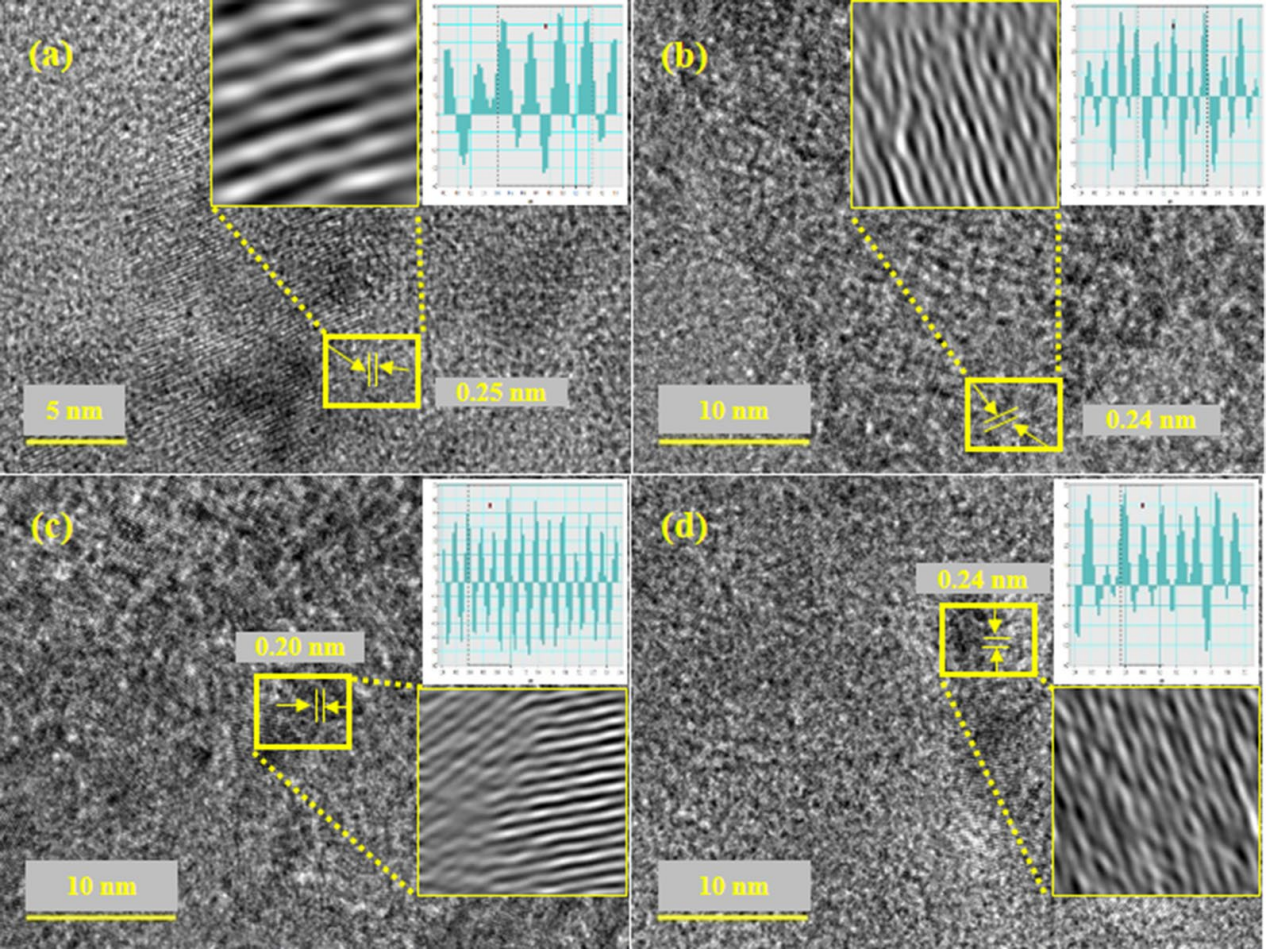
d-spacing calculated using HR-TEM images of GO-MgO (**a**–**d**) with GO content (0, 0.01, 0.03, 0.05)

Interlayer d-spacing was measured with HR-TEM images using Gatan software as illustrated in Fig. 6a–d. The d-spacing values for various concentrations (0:1, 0.01:1, 0.03:1 and 0.05:1) of GO-doped MgO were calculated as 0.250, 0.240, 0.20 and 0.24 nm, respectively, assigned to (200) plane of MgO (Fig. 6a–d) as synchronized with XRD results. Moreover, change in d-spacing values has been assigned to GO doping into MgO lattices.

**Fig. 7:**
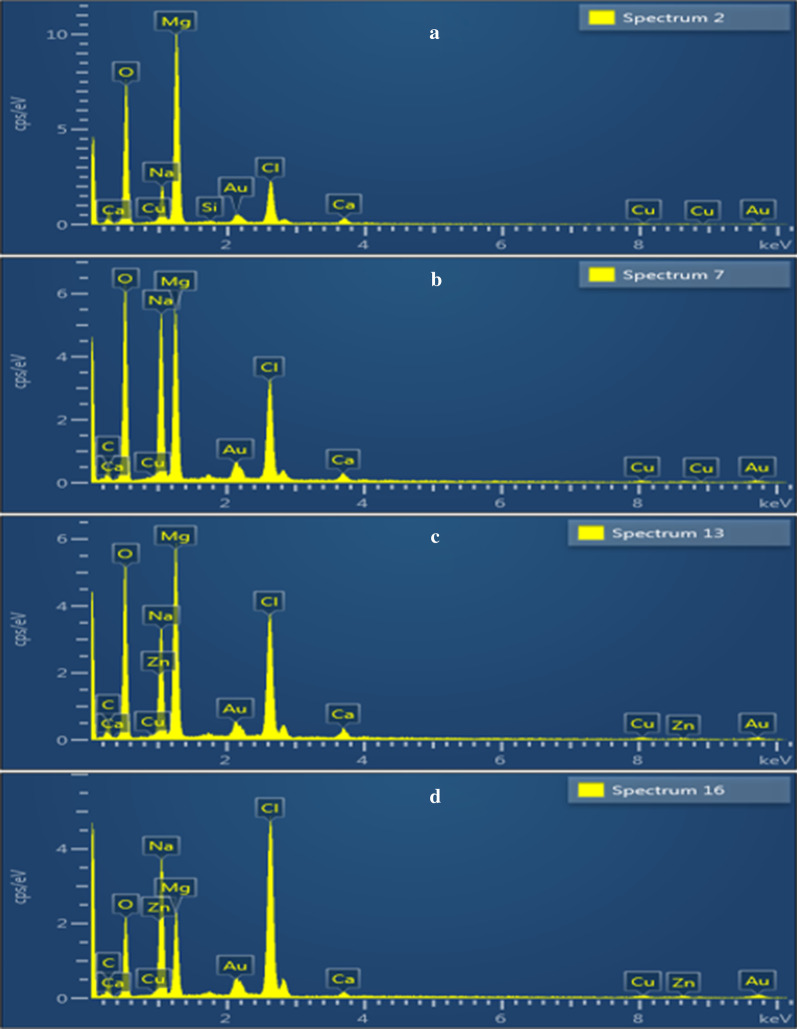
SEM–EDS analysis of GO-doped MgO (**a**–**d**) with GO content (0, 0.01, 0.03 and 0.05), respectively

SEM–EDS analysis disclosed the detailed information on sample surface regarding its elemental composition. EDS spectra of MgO and various ratios (0.01:1, 0.03:1 and 0.05:1) of GO into MgO are expressed in Fig. 7a–d, respectively. The presence of magnesium (Mg) and oxygen (O) detected in Fig. 7a are confirmation to MgO formation. Carbon (C) peak was ascribed to GO nanosheets, while Na elemental peak in samples was observed due to use of NaOH during synthesis process to maintain pH.

**Fig. 8:**
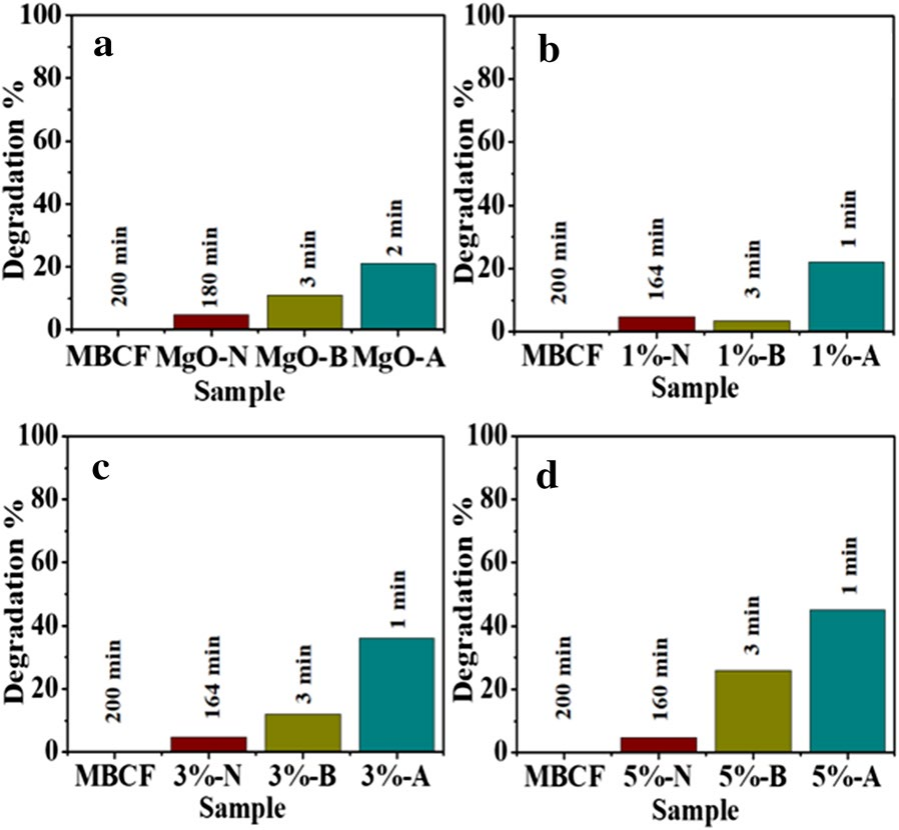
**a**–**d** Catalysis of blank MBCF and GO-doped MgO in different dye medium [neutral (N), basic (B) and acidic (A)], respectively

To test the catalytic activity of GO-doped MgO, UV–Vis absorption spectra attained upon reference sample degradation (MBCF) were used. Reducing capacity of NaBH_4_ with MBCF was not significantly affected after 200 min as demonstrated in Fig. 8a–d. In neutral condition, MBCF (3 mL) solution added into 400 μL NaBH_4_ and 3 mL samples within 180–160 min showed limited reduction (4.8% degradation) for undoped and doped samples. Moreover, in basic activity for samples (0:1, 0.01:1, 0.03:1, 0.05:1) resulted 11, 3.5, 12, 26% degradation in 3 min, respectively (Fig. 8a–d). Highest catalytic function was obtained in acidic solution with higher concentration (0.05) of GO in MgO nanostructures showing 45% degradation in 1 min as depicted in Fig. 8d.

During catalysis, reduction in MBCF in the presence of NaBH_4_, synthesized materials act as electron relay such that transfer of electron from BH_4_^−^ ions (donor) to MBCF (acceptor) results in the reduction in dye [57]. Abundant active sites of nanostructures enhanced adsorption for BH_4_^−^ ions and dye molecules to react with each other. Solution pH also affects performance of degradation. For acidic medium (H_2_SO_4_), catalytic activity enhanced which is attributed to increased generation of H^+^ ions offered to be adsorbed on the surface of nanostructure. Upon addition of NaOH for basic medium, the number of hydroxyl groups increases leading to oxidation of reduced products and decrease in catalytic activity. Results showed that dye degradation by nanostructures under acidic medium was much higher compared to basic condition.

The in vitro antibacterial efficacy of pure and GO-doped MgO was performed against G −ve and +ve isolates by well diffusion assay (Table 1). Results depicted improved microbicidal action and synergism of GO-MgO for *E. coli* in comparison with *S. aureus*, see Table 1. Significant (*p* < 0.05) inhibition areas were recorded as (1.55–4.75 mm) and (2.10–4.85 mm) for *E. coli* at minimum and maximum dose, respectively, and (1.30–4.00 mm) for *S. aureus* at high dose. All concentrations showed zero antibacterial efficacy for *S. aureus* at low dose. Results comparison proceeded with −ve control DI water (0 mm) and +ve control ciprofloxacin (7.15 mm) and (11.25 mm) inhibition areas for *E. coli* and *S. aureus*, respectively.

**Table 1:** Antibacterial activity of GO-doped MgO

Sample	Inhibition zone^a^ (mm)		Inhibition zone^b^ (mm)	
	0.5 mg/50 μL	1.0 mg/50 μL	0.5 mg/50 μL	1.0 mg/50 μL
MgO	0	1.30	1.55	2.10
GO/MgO 1%	0	2.25	2.60	3.35
GO/MgO 3%	0	3.15	3.80	4.40
GO/MgO 5%	0	4.00	4.75	4.85
Ciprofloxacin	11.25	11.25	7.15	7.15
DI water	0	0	0	0

^a^Inhibition zones diameters (mm) for *S. aureus*

^b^Values of zones of inhibition for *E. coli*

Overall, doped nanostructures revealed zero bactericidal activity towards G +ve in low dose, while effectiveness towards G –ve was substantial (*p* < 0.05) relative to G +ve in doped content.

**Fig. 9:**
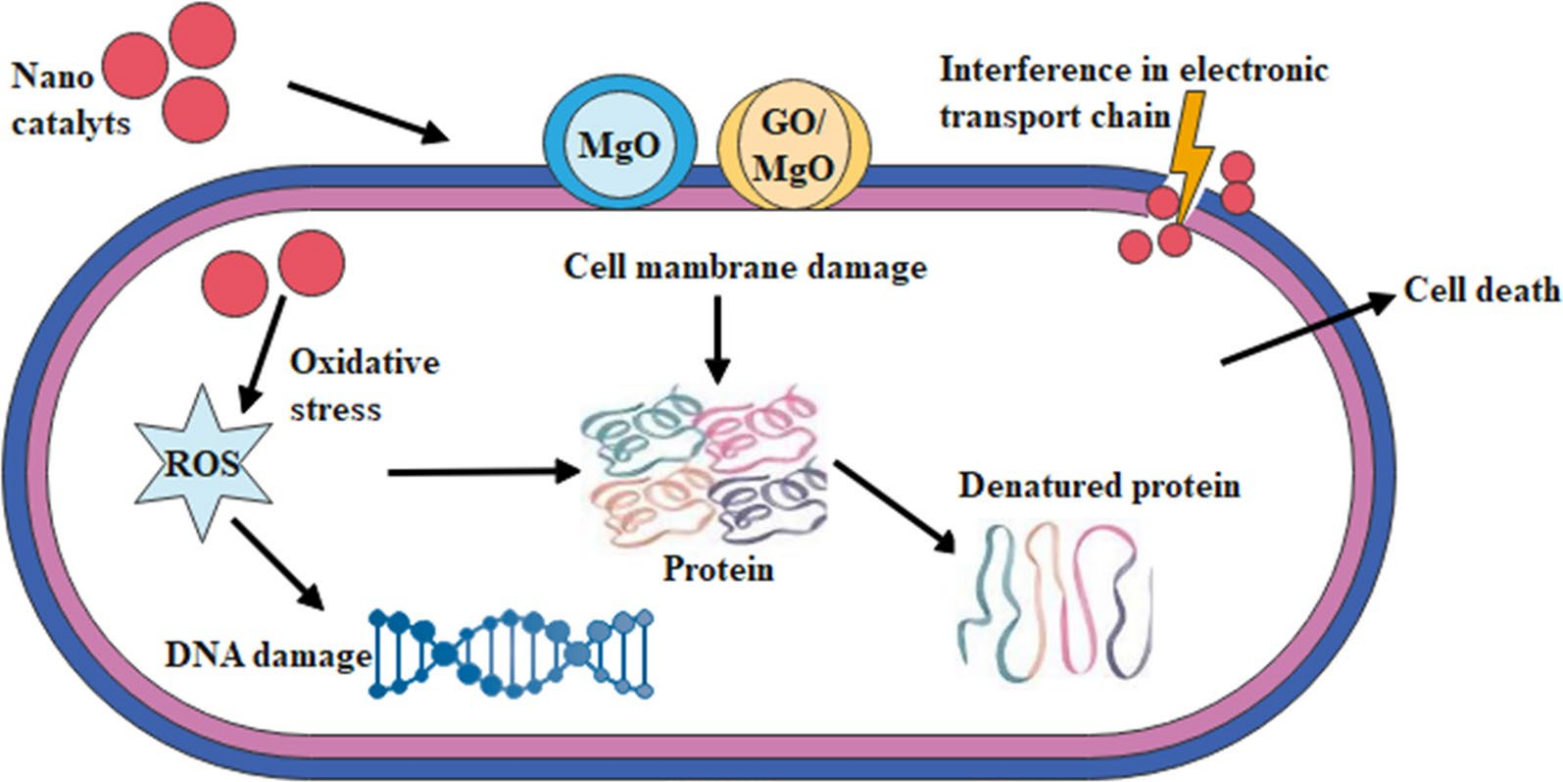
Proposed mechanism of bactericidal activity GO-MgO nanostructures

The oxidative stresses of engineered nanostructures rely on diverse factors such as shape, size and concentration of nanoparticles which play an important role in antibacterial action [58]. Small nano-sized materials efficiently produce reactive oxygen species (ROS) causing damage of bacteria cell membrane and extrusion of cytoplasm, resulting bursting of bacteria, see Fig. 9 [59]. Secondly, significant nanomaterial cationic interference with negative bacterial cell membrane fragments result in collapse. Antibacterial activity of MgO nanoparticle enhanced against E. coli, which was on account of the generation of a large amount of O_2_^−^ and may be lipid peroxidation and ROS [60]. In dopant sample, antibacterial activity increased due to increasing GO concentration.

**Table:** 

Samples	Control sample band gap (eV)	Decreasing in band gap (eV)	References
GO-doped ZnO	3.6	2.9	[61]
Ag-doped GO	4.10	3.50	[2]
GO-doped ZnO	3.10	2.98	[62]
GO-doped MgO	5.0	4.8	Present study

## Conclusion

In the present work, GO-doped MgO nanostructures were successfully synthesized with chemical precipitation route. The cubic structure of MgO was observed using XRD technique and confirmed with HRTEM. Molecular bonding of Mg-O with various functional groups and characteristic transmittance peaks of MgO around 443 cm^−1^ in fingerprint region were recorded using FTIR. The absorption increased upon doping, which introduced redshift at higher concentrations of GO. Calculated band gap using Tauc plot from absorption spectra of doped MgO found to be 4.8 eV (high concentration) comparative to MgO (5.0 eV) was ascribed to redshift in absorption upon doping. Cubic and hexagon morphology of nanostructures was observed in MgO and growth of rod-like structures was observed upon doping with a decrease in diameter of hexagons in HR-TEM. Furthermore, average d-spacing (0.23 nm) from HRTEM with Gatan software are well matched with XRD. EDS analysis revealed elemental composition that showed confirmation of Mg, O with GO doping. With the incorporation of GO, intensity of PL decreased from 414 nm accompanied with a blueshift indicating low recombination rate of excitons. The presence of D and G band (at 1338, 1598 cm^−1^, respectively) associated with *sp*^3^ and *sp*^2^ C atom were verified with Raman. Catalytic activity was assessed and highest dye degradation of about 45% was attained in acidic condition by 0.05 GO-MgO. In addition, experimental results showed enhanced bactericidal efficacy of GO-MgO against G –ve (*E. coli*) relative to G +ve (*S. aureus*). In addition, synergism of GO-MgO showed enhanced bactericidal efficacy against G –ve (*E. coli*) compared to G +ve (*S. aureus*). This study explored the dopant-dependent properties of MgO nanocomposites that can be employed to clean industrial polluted water and in antimicrobial applications for environmental remediation.

## Data Availability

All data are fully available without restriction.

## Abbreviations

EDS: Energy-dispersive X-ray spectroscopy
FTIR: Fourier transform infrared spectroscopy
FESEM: Field emission scanning electron microscopy
G +ve: Gram-positive
G –ve: Gram negative
GO: Graphene
HR-TEM: High resolution transmission electron microscopy
JCPDS: Joint committee on powder diffraction standards
MgO: Magnesium oxide
UV–Vis: Ultra-violet–visible spectroscopy
XRD: X-ray diffraction
